# 2017 FDA Peptide Harvest

**DOI:** 10.3390/ph11020042

**Published:** 2018-05-07

**Authors:** Othman Al Musaimi, Danah Al Shaer, Beatriz G. de la Torre, Fernando Albericio

**Affiliations:** 1 College of Health Sciences, University of KwaZulu-Natal, Durban 4000, South Africa; musamiau@gmail.com (O.A.M.); danah.shaer@gmail.com (D.A.S.); 2 School of Chemistry, University of KwaZulu-Natal, Durban 4001, South Africa; 3 KRISP, College of Health Sciences, University of KwaZulu-Natal, Durban 4001, South Africa; 4 CIBER-BBN, Networking Centre on Bioengineering, Biomaterials and Nanomedicine, University of Barcelona, 08028 Barcelona, Spain; 5 Department of Organic Chemistry, University of Barcelona, 08028 Barcelona, Spain

**Keywords:** pharmaceutical market, drugs, drug discovery, solid-phase peptide synthesis

## Abstract

2017 was an excellent year in terms of new drugs (chemical entities and biologics) approved by the FDA, with a total of 46. In turn, one of the highlights was the number of peptides (six) included in this list. Here, the six peptides are analyzed in terms of chemical structure, synthetic strategy used for their production, source, therapeutic use, and mode of action.

## 1. Introduction

**Figure 1:**
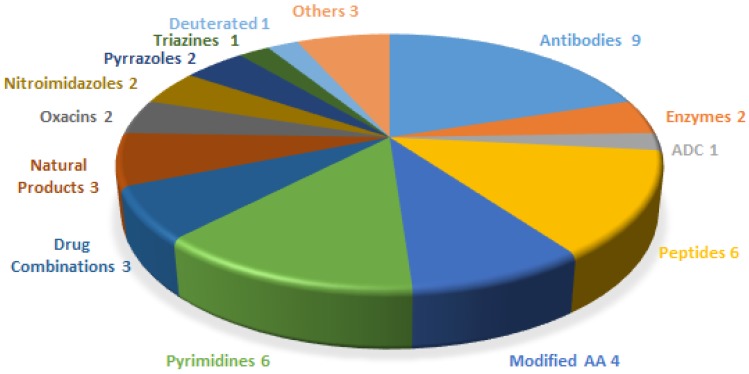
New drugs approved by the FDA in 2017 and classified on the basis of chemical structure.

The financial investment associated with the pharmaceutical industry is one of the largest in the industrial sector—surpassed only by the telecommunications sector. However, the number of new products (drugs) entering the market each year is relatively low. In this context, 2017 was an exceptional year, in that 46 new drugs were approved by the US Food and Drug Administration (FDA) [1]—the highest figure in the last twenty-five years. Drugs can be broadly divided into two main groups. The first encompasses biologics (12 approved in 2017, accounting for 25% of the total number of drugs approved), which are prepared by means of biotechnological techniques. The second group comprises chemical entities (34 approved in 2017), which are prepared using chemical synthesis [2]. In turn, chemical entities can be grouped into two categories, the so-called small molecules, which also include some natural products, and TIDES (peptides and oligonucleotides). Figure 1 shows the drugs approved by the FDA in 2017 and classified on the basis of their chemical structure. Thus, in a clockwise direction, biologics (antibodies, enzymes, and antibodies drug conjugates) appear first, followed by peptides, modified amino acids, and more traditional small molecules. 

Along a similar line, 2017 was an excellent year for peptides, with the FDA approving five peptides and one peptidomimetic, which together accounted for 13% of the drugs accepted that year. 

However, the 2017 figures should be interpreted with care. They cannot be taken as a trend since the arrival of a drug onto the market involves many unpredictable variables.

From a structural point of view, the six peptides in the 2017 harvest show almost the full range of diversity, probably lacking only a homodetic cyclic peptide and/or a cyclodepsipeptide. In this regard, in addition to a peptidomimetic macimorelin (MacrilenTM), the 2017 harvest included two linear peptides angiotensin II (GiaprezaTM) and abaloparatide (TymlosTM) with 8 and 34 amino acids, respectively, and a peptide plecanatide (TrulanceTM) containing two disulphide bridges. It also included the following two unique branched peptides: semaglutide (OzempicTM) with a chain pending at a Lys residue, which contains two mini-PEG amino acids, a Glu residue linked to the chain through the ω-carboxylic group, and a C18 diacid; and etelcalcetide (ParsabivTM), which is formed by a linear chain of seven D-amino acids with a disulphide bridge between a D-Cys with a single L-Cys. Interestingly, three of these peptides (macimorelin, abaloparatide, and semaglutide) contain a residue of the non-proteinogenic aminoisobutyric (Aib) acid, with the purpose of conferring stability against peptidases.

Only one of these peptides have been developed by two so-called big pharmas (semaglutide by Novo Nordisk A/S) and the rest by biotech companies. Macimorelin had its roots in Fehrentz and Martinez’s group at the University of Montpellier (France). The five peptides other than macimorelin were produced using the solid-phase technique. 

## 2. Plecanatide (Trulance)

**Figure 2:**
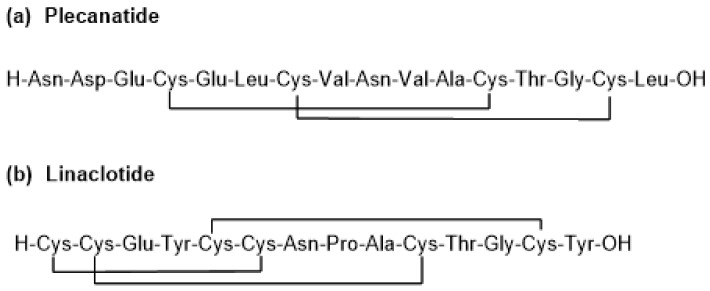
Structure of (**a**) plecanatide and (**b**) the related linaclotide.

This peptide has a linear sequence of 16 amino acids with two disulphide bridges pairing Cys 4 with Cys12 and Cys7 with Cys15. Its C-terminal residue is in acid form (molecular weight of 1681.9 Da) (Figure 2a). It is manufactured using solid-phase technique.

Plecanatide differs from uroguanylin (the endogenous counterpart of plecanatide) only in the replacement of Asp3 by Glu3 [3].

It was developed by Synergy Pharmaceuticals (New York City, NY, USA) and was approved by the FDA on 7 January 2017 for the treatment of chronic idiopathic constipation (CIC) and irritable bowel syndrome with constipation (IBS-C) [4]. Plecanatide is an agonist of guanylate cyclase-C, it increases intestinal transit and fluid through a build-up of guanosine 3′,5′-cyclic monophosphate (cGMP) [5] and has a similar mode of action as linaclotide (Constella-Linzess) (Figure 2b), which is a 14-amino acid peptide containing three disulphide bridges which are located between Cys1 and Cys6, between Cys2 and Cys10, and between Cys5 and Cys13. Linaclotide was approved by the FDA in 2012 [6].

Plecanatide draws water into the gastrointestinal (GI) tract, thereby softening stool and encouraging its natural passage. It activates guanylate cyclase-C (GC-C) on endothelial cells within the GI [7]. The pH-dependent activation of GC-C receptors by plecanatide (as it has the acidic residues Asp2 and Glu3) may promote bowel movements without causing severe diarrhea [3,7]. Furthermore, in molecular dynamics simulations, plecanatide showed optimal activity at pH 5, indicating that the proximal intestine (pH 5–6) is the ideal site of action [8].

The activation of GC-C catalyzes the production of the second messenger cGMP, which leads to the protein kinase A (PKA)- and protein kinase G II (PKGII)-mediated phosphorylation of the cystic fibrosis transmembrane conductance regulator (CFTR) protein [9]. Upon activation, CFTR secretes chloride (Cl−) and bicarbonate (HCO3−) into the GI tract lumen, followed by the passive secretion of positively charged sodium ions into the lumen, and water follows by osmosis [10].

In the GI tract, plecanatide is metabolized by intestinal enzymes. The excretion of plecanatide has not been studied in humans [3].

Plecanatide is administered orally as is linaclotide. These two examples showcase the feasibility of the oral administration of peptides.

## 3. Etelcalcetide (Parsabiv)

**Figure 3:**
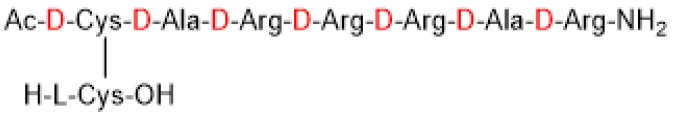
Structure of etelcalcetide. Amino acids of D configuration are shown in red.

This is an octapeptide formed by a linear chain of seven D-amino acids containing a D-Cys, which is linked through a disulphide bridge to an L-Cys. The C-terminal residue is in amide form (molecular weight of 1048.3 Da), and it is manufactured using a solid-phase technique (Figure 3). The presence of amino acids in D configuration confers the peptide chain resistance to proteolytic degradation. The presence of disulphide bonds facilitates the biotransformation process, especially with endogenous thiols in blood, and this is considered a main metabolic pathway of etelcalcetide [11,12,13].

Etelcalcetide was developed by KAI Pharmaceuticals Inc. (South of San Francisco, CA, USA), a wholly subsidiary of Amgen Inc. (Thousand Oaks, CA, USA) and approved by the FDA on 7 February 2017 [14]. It is used for the treatment of secondary hyperparathyroidism (SHPT) in chronic kidney disease (CKD) in adult patients on hemodialysis [11,12,15,16,17,18]. Cardiovascular calcination is common in CKD patients, and it occurs as a result of impaired mineral homeostasis and secondary hyperparathyroidism [16]. As a calcimimetic agent, etelcalcetide binds to the calcium-sensing receptor (CaSR) through a disulphide bridge between the D-Cys of the etelcalcetide molecule and L-Cys of the CaSRs, thereby enhancing activation of the receptor by means of extracellular calcium. Accordingly, activation of CaSRs on parathyroid chief cells decreases the secretion of parathyroid hormone (PTH), as well as fibroblast growth factor-23 (FGF23), which is stimulated by PTH [12,13,15,16,17,18,19,20,21]. Furthermore, etelcalcetide decreases phosphorus in the blood. Interestingly, high blood phosphorus occurs in vascular calcification [16].

A serious side effect of etelcalcetide is that it reduces serum calcium levels, which might lead to hypocalcemia. Therefore, monitoring serum calcium (after etelcalcetide dosing is initiated), as well as PTH, is deemed necessary [13,15,21]. Etelcalcetide can cause vomiting and nausea [11,13,20,21].

## 4. Abaloparatide (Tymlos)

**Figure 4:**
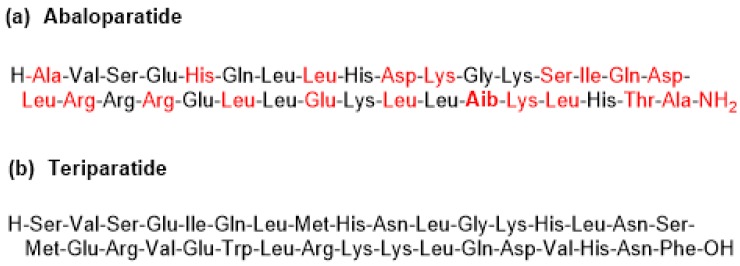
Structure of (**a**) abaloparatide and (**b**) teriparatide. The residues modified are shown in red. The non-proteinogenic amino acid Aib is shown in bold.

This is a linear C-terminal amide peptide that contains 34 amino acids. The C-terminal residue is in amide form (molecular weight of 3960.7 Da) (Figure 4a). It is manufactured by a hybrid solution–solid phase approach.

Abaloparatide can be considered a second generation teriparatide (Forteo) (Figure 4b), which is a recombinant form of PTH (84 amino acids), formed by the N-terminal fragment (34 amino acids) of PTH. Abaloparatide contains exactly the same number of amino acids as teriparatide but has multiple substitutions. It has 41% homology with teriparatide [22]. Interestingly, abaloparatide has an Aib residue at position 29.

Abaloparatide was developed by the biotech company Radius Health, Inc. (Waltham, MA, USA) and approved by the FDA on 28 April 2017 [23].

Abaloparatide works as an anabolic (bone-growing) agent through the selective activation of the parathyroid hormone 1 receptor (PTH1R), a G protein-coupled receptor (GPCR) expressed in osteoblasts and osteocytes [22]. This receptor can be present in two distinct conformation states (R0 and RG), which differ in their signaling response. Ligands that bind selectively to the RG state result in a shorter signaling response, whereas those that bind selectively to the R0 state lead to a prolonged response [24]. Abaloparatide preferentially binds to the RG state of PTH1R, which in turn elicits a transient downstream cyclic AMP signaling response towards a more anabolic signaling pathway [22,24].

Abaloparatide outperforms teriparatide as an anabolic agent, as shown by the increased messenger ribonucleic acid (RNA) expression level for the receptor activator of nuclear factor kappa-B ligand (RANKL) and macrophage colony-stimulating factor in a human osteoblastic cell line. Although the molecular mechanisms underlying the differences between abaloparatide and teriparatide are not well understood, they may be related to conformational differences that determine the affinities of the drugs for PTHR1 [22].

## 5. Semaglutide (Ozempic)

**Figure 5:**
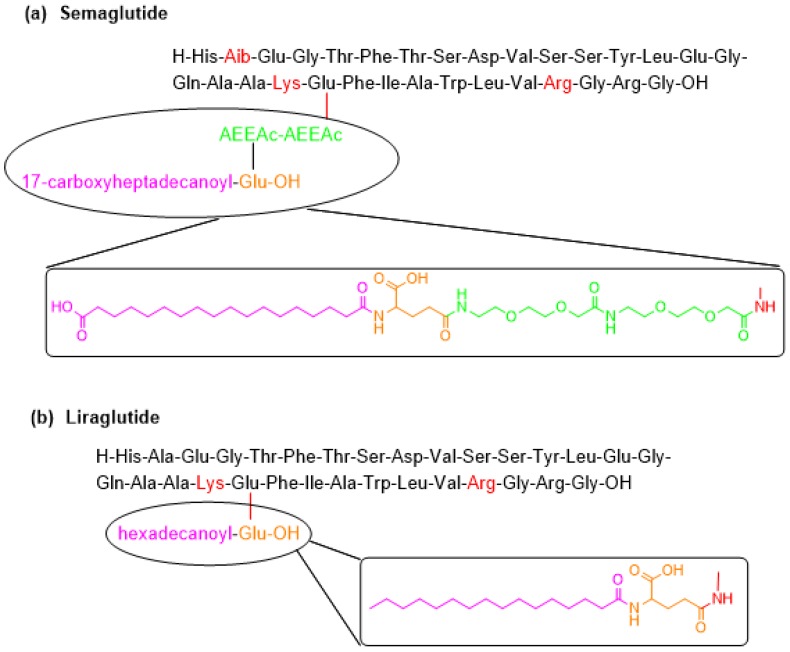
Structure of (**a**) semaglutide and (**b**) its related liraglutide. Changes in structure with respect to GLP-1 (7-37) are shown in color.

Semaglutide contains a linear sequence of 31 amino acids, with a moiety pending from the ε-amino function of Lys20 (the numeration of the amino acids in semaglutide is done by taking as reference the numeration in the parent peptide GLP-1), which contains a Glu residue linked to the ε-amino group of Lys side-chain through the γ-carboxylic group, two mini-PEG amino acids [8-amino-3,6-dioxaoctanoic acid (ADO)] and a C18 diacid (Figure 5a). The C-terminal is in the form of a carboxylic acid (molecular weight of 4113.6 Da). It is manufactured using a solid-phase approach.

Semaglutide is a member of the glucagon like peptide-1 (GLP-1) family, derived from the GLP-1 (sequence 7-37), and can be considered the second generation of liraglutide (Figure 5b), which was accepted by the FDA in 2010 [25]. Liraglutide differs from GLP-1 (7-37) (Figure 5b) in the presence of Arg in position 34 instead of Lys and of a moiety at Lys20, which is a reduced version of the one in semaglutide. When comparing the structures of semaglutide and liraglutide, in addition to the pending moiety, semaglutide has Aib instead of Ala in position 8, thereby reducing the susceptibility of semaglutide to degradation by dipeptidyl peptidase-4 [26,27,28]. Both semaglutide and liraglutide were developed by Novo Nordisk A/S (Måløv, Denmark). Semaglutide was approved by the FDA on 21 December 2017 [29].

The GLP-1 family stimulates insulin and decreases glucagon secretion. However, GLP-1 has a short half-life (1–2 min) as a result of proteolytic degradation, thus hindering its use as a potential treatment for type 2 diabetes [27]. Liraglutide is the first once-daily glucagon-like peptide-1 analogue designed to resist enzymatic degradation and thus have a longer half-life [26,27,30]. The presence of the 17-carboxyheptadecanoyl fatty acid moiety results in its binding to human albumin, which is responsible for the longer-acting activity of liraglutide in comparison with other members of the same family. The rationale behind the design of semaglutide, which allows once-weekly administration, is to increase the affinity of the pending fatty acid moiety for albumin. Moreover, semaglutide has no serious adverse effects, only some mild gastrointestinal disorders [27].

## 6. Macimorelin (Macrilen)

**Figure 6:**
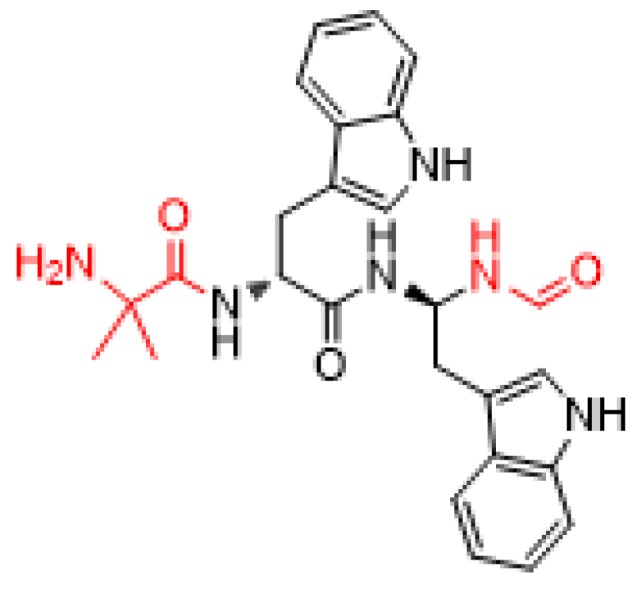
Structure of macimorlein. Modifications with respect to a tripeptide are shown in red.

Macimorelin is a small pseudopeptide formed by three residues: Aib as N-terminus, D-Trp at the central position, and a mimetic of D-Trp—a gem diamino moiety—which is formylated at its N-terminus (Figure 6) (molecular weight of 474.6 Da). It is prepared by solution synthesis.

Macimorelin was discovered by Fehrentz and Martinez’s group at the University of Montpellier [31] and developed by the biotech company Aeterna Zentaris GmbH (Frankfurt, Germany). It was approved by the FDA on 20 December 2017 [32]. Administered orally, it is used for the diagnosis of adult growth hormone deficiency (AGHD).

Macimorelin acts as a growth hormone secretagogue (GHS) mimicking ghrelin, which is a 28-amino acid peptide produced by the stomach and is the endogenous ligand for this GHS receptor [31,33,34,35,36]. In addition to being orally bioavailable, macimorelin is selective, tolerable, and also safe, with only mild adverse effects—such as an unpleasant taste—being reported [34,35,37].

By acting in an almost identical manner to ghrelin [37], macimorelin outperforms other GHS such as the expensive recombinant human GH.

## 7. Angiotensin II (Giapreza)

**Figure 7:**
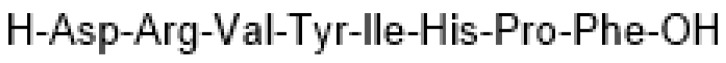
Structure of angiotensin II.

Angiotensin II is a simple linear octapeptide formed by natural amino acids of the L series and its structure is identical to the human hormone of the same name. The C-terminal is in the form of carboxylic acid (molecular weight of 1046.2 Da) (Figure 7). It is manufactured using the solid-phase approach.

Angiotensin II was developed by a biotech company, La Jolla Pharmaceutical Company (San Diego, CA, USA), and approved by the FDA on 21 December 2017 [38]. It is recommended as a vasoconstrictor to increase blood pressure in adults with septic or other distributive shock. It is administered intravenously because its half-life is approximately 30 s.

Angiotensin II is related to the renin–angiotensin System (RAS). From a drug discovery perspective, it can be considered unique among the drugs approved by the FDA in recent years. Its roots can be found in the last part of the XIX century, when Tigerstedt and Bergman discovered the effect of renal extracts on arterial pressure [39]. In the 1930s, two independent groups, one in Argentina with Leloir, Houssay, Fernandez Braun, among others, and that of Page in the US, discovered that RAS is the hormone system that regulates blood pressure and fluid balance [40]. In 1957, again two groups—one in the US (Schwarz and colleagues) [41] and the second in Switzerland (GIBA Geigy) [42]—described the first synthesis of angiotensin II. Seventy years after its first synthesis, this octapeptide reached the market. 

Angiotensin II is formed after the removal of two C-terminal residues of angiotensin I by the angiotensin-converting enzyme (ACE). In turn, angiotensin I is the N-terminal part of angiotensinogen, an α-2-globulin produced constitutively and released into the circulation mainly by the liver.

As a summary, Table 1 shows the six peptides approved by the FDA in 2017 highlighting several parameters (chemical modification, source, therapeutic use, mode of action, and administration) that have been key to their development.

Finally, it is important to recall the trend of the peptide market. This market was worth US$5.3 billion in 2003, rising to US$8 billion in 2005 and US$14.1 billion in 2011, and it is expected to reach a value of US$25.4 billion and US$46.6 billion by the end of 2018 and 2024, respectively [43,44,45]. Furthermore, there are currently hundreds of peptides in preclinical testing stages and around 150 peptides in clinical development. Many of these molecules are showing a promising therapeutic impact [36,43,46,47,48].

It is to be hoped that the coming years will bring about the approval of a similar number of peptides to those accepted by the FDA in 2017 and that the trends of the market in terms of peptide development continue, thus making these molecules one of the best options to treat many diseases.

## Figures and Tables

**Table 1:** Summary of the peptides approved by the FDA in 2017.

Generic Name (Trade Name)	Company	Mode of Action	Therapeutic Use	Administration
Plecanatide (Trulance)	Synergy Pharmaceuticals, Inc.	Activation of guanylate cyclase-C	Gastrointestinal laxative	Oral
Etelcalcetide (Parsabiv)	KAI Pharmaceuticals, Inc. *	Activation of CaSR on parathyroid chief cells	Secondary hyperpara-thyroidism in adult patients with chronic kidney disease on hemodialysis	IV
Abaloparatide (Tymlos)	Radius Health, Inc.	Selective activation of the parathyroid hormone 1 receptor	Osteoporosis	SC
Semaglutide (Ozempic)	Novo Nordisk, Inc.	Acts as a Glucagon-like Peptide-1 agonist	Treatment of type 2 diabetes mellitus	SC
Macimorelin (Macrilen)	Aeterna Zentaris, Inc.	Mimic the endogenous ligand for the secretagogue (Ghrelin)	For the diagnosis of adult growth hormone deficiency	Oral
Angiotensin II (Giapreza)	La Jolla Pharm Co.	Acts on the CNS to increase ADH production	Control of blood pressure in adults with sepsis or other critical conditions	IV

* Wholly owned subsidiary of Amgen, Inc.; IV: intra venous; SC: subcutaneous.
